# Neural correlates of abnormal auditory feedback processing during speech production in Alzheimer’s disease

**DOI:** 10.1038/s41598-019-41794-x

**Published:** 2019-04-05

**Authors:** Kamalini G. Ranasinghe, Hardik Kothare, Naomi Kort, Leighton B. Hinkley, Alexander J. Beagle, Danielle Mizuiri, Susanne M. Honma, Richard Lee, Bruce L. Miller, Maria Luisa Gorno-Tempini, Keith A. Vossel, John F. Houde, Srikantan S. Nagarajan

**Affiliations:** 1 Memory and Aging Center, Department of Neurology, University of California San Francisco, San Francisco, CA 94158 USA; 2 Speech Neuroscience Laboratory, Department of Otolaryngology - Head and Neck Surgery, University of California San Francisco, San Francisco, CA 94143 USA; 3 Biomagnetic Imaging Laboratory, Department of Radiology and Biomedical Imaging, University of California San Francisco, San Francisco, CA 94143 USA; 4 UC Berkeley - UCSF, Graduate Program in Bioengineering, San Francisco, CA, USA; 5 N. Bud Grossman Center for Memory Research and Care, Institute for Translational Neuroscience, and Department of Neurology, University of Minnesota, Minneapolis, MN 55455 USA

## Abstract

Accurate integration of sensory inputs and motor commands is essential to achieve successful behavioral goals. A robust model of sensorimotor integration is the pitch perturbation response, in which speakers respond rapidly to shifts of the pitch in their auditory feedback. In a previous study, we demonstrated abnormal sensorimotor integration in patients with Alzheimer’s disease (AD) with an abnormally enhanced behavioral response to pitch perturbation. Here we examine the neural correlates of the abnormal pitch perturbation response in AD patients, using magnetoencephalographic imaging. The participants phonated the vowel /α/ while a real-time signal processor briefly perturbed the pitch (100 cents, 400 ms) of their auditory feedback. We examined the high-gamma band (65–150 Hz) responses during this task. AD patients showed significantly reduced left prefrontal activity during the early phase of perturbation and increased right middle temporal activity during the later phase of perturbation, compared to controls. Activity in these brain regions significantly correlated with the behavioral response. These results demonstrate that impaired prefrontal modulation of speech-motor-control network and additional recruitment of right temporal regions are significant mediators of aberrant sensorimotor integration in patients with AD. The abnormal neural integration mechanisms signify the contribution of cortical network dysfunction to cognitive and behavioral deficits in AD.

## Introduction

**Figure 1:**
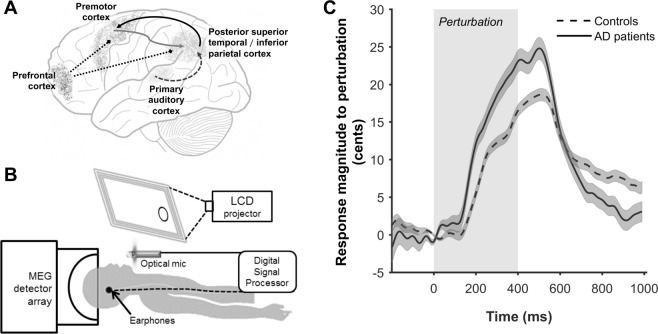
Cortical circuits of speech motor control, schematic of the experimental setup and the behavioral response to pitch perturbation. (**A**) Anatomical locations of candidate cortical areas are depicted on a schematic brain diagram. The arrows indicate auditory feedback control pathways where feedback predictions (grey arrow) are compared with incoming feedback from primary auditory cortices (dashed arrow) in the posterior superior temporal/inferior parietal cortices to generate feedback corrections (black arrows). These key processing nodes (premotor cortex and posterior superior temporal/inferior parietal cortex) are modulated by prefrontal cortex (dotted lines). The experimental setup (**B**) illustrates the participant as they are lying supine in the MEG scanner. The subject speaks into an optical microphone and receives auditory feedback through a set of air-tube earphones. Their speech is passed through a digital signal processor, which generates the pitch-shifted audio feedback stimulus at a jittered delay after speech onset. The LCD panel directly in front of the subject gives a visual clue (a clearly identifiable colored circle), which prompts the participant to start speaking. (**C**) The magnitude of vocal response to perturbations of 100 cents for controls and patients with AD. Dark lines indicate the mean response, and the shaded areas indicate standard error, across the trials per each group. The time axis is time-locked to perturbation onset (0 ms). Grey shaded area indicates the duration of perturbation. Abbreviations: AD = Alzheimer’s disease. LCD = liquid crystal display; MEG = magnetoencephalography.

From reaching and grasping to speaking and singing, motor skills require accurate integration of sensory and motor signals to achieve target behavioral goals. The motor commands to achieve a target also generate an efferent-copy of the sensory consequences^1^. These internal predictions are constantly compared against sensory feedback signals from the periphery, and the motor output is adjusted to correct for feedback errors. Speaking is a particularly good example where sensory processing of auditory feedback is successfully integrated to modulate speech output^2^. Current models of speech production posit that, during speaking, the higher frontal cortex responds by activating a speech motor control network—a distributed network including primary and higher order auditory cortices and premotor cortex^3–5^ (Fig. 1A). Speech output is continually adjusted as the incoming feedback from both auditory and somatosensory signals are being compared with the sensory predictions derived from motor efferent-copy^6–10^. A well-studied experimental paradigm to test sensorimotor integration during speech production is the pitch perturbation response. In such experiments, speakers phonate a vowel while a digital audio processing system perturbs how they hear the pitch of their own speech. The feedback perturbations cause speakers to compensate for the applied pitch shift: e.g., if the pitch of the audio feedback is lowered speakers will raise their pitch^6^. In a previous study we showed that patients with Alzheimer’s disease (AD) have an abnormally enhanced behavioral response to altered pitch in their auditory feedback, indicating abnormal sensorimotor integration processes in AD^5^. In this study, we sought to investigate the specific neural mechanisms underlying the abnormal pitch perturbation response in AD patients.

The defining phenotypic characteristic of AD is progressive loss of memory, executive, language and visuospatial abilities. Given that patients with AD have relatively low incidence of purely sensory and motor deficits in neurological exam, traditionally, sensorimotor dysfunctions have been held at a low profile in AD^11^. However, from a neural network perspective, processing of sensory information and generation of behavioral motor commands as well as the neural processes of cognitive functions are fundamentally interconnected^12^. For instance, impairment in sensorimotor integration processes may compromise the function of a particular cognitive domain, while an abnormal cognitive process may reduce its modulatory effect on sensory or motor pathways. Intrinsically connected brain networks provide the functional architecture that support the processing of sensory inputs and motor outputs as well as modulation through top-down regulatory mechanisms. Indeed, a growing body of evidence implicate dysfunction within large-scale neural networks as the basis for the loss of cognitive abilities in AD^13–15^. As such, an abnormally enhanced behavioral pitch perturbation response in AD patients may either result from abnormal sensorimotor processing mechanisms within the speech-motor control network itself, or as a result of failed regulatory mechanisms across cognitive systems. Evidence for interconnections between speech-motor control network and other cognitive systems come from studies of healthy participants where auditory attention and auditory working memory have been shown to modulate the behavioral and neural correlates of speech motor control^16–20^. Moreover, in our previous behavioral experiment we found that the degree of enhanced pitch perturbation response was significantly correlated with executive dysfunction in AD patients^5^. Hence, it is reasonable to hypothesize that AD patients would likely exhibit deficits in frontally mediated regulatory mechanisms on speech motor control network, although this hypothesis has never been directly tested.

Our study participants included patients with AD and age-matched controls and consisted a partially overlapping cohort from the previously reported behavioral experiment (see methods for details). The pitch perturbation task paradigm was based on the altered pitch of the subjects’ auditory feedback during speaking and was the same as detailed in previous reports^5^. Using magnetoencephalographic imaging (MEGI) we examined the high-gamma band (65–150 Hz) activity during pitch perturbation and investigated neural-behavioral correlates to identify the specific mechanisms driving the abnormal pitch perturbation response in AD^21,22^. Based on reasoning stated in the previous paragraph, we hypothesized that patients with AD would show reduced activity patterns in the prefrontal cortices during pitch perturbation response than healthy controls indicating abnormal frontally mediated modulatory effects, and that such altered neural activity patterns would account for the enhanced behavioral response in patients with AD.

## Methods

### Participants

Sixteen patients meeting the diagnostic criteria for AD^23^ and 13 age-matched healthy volunteers participated in the study. Fourteen out of 16 AD patients in the current study overlapped with the cohort of 19 patients that participated in the behavioral experiment reported in our previous paper^5^. The 13 control participants in the current study consisted of a subset of the previous control cohort of 16 participants. Altogether, two patients with AD reported in this study did not take part in the experiments reported in our earlier paper, while eight participants who exclusively contributed to behavioral data that was reported in the earlier paper are not included in the current study. The behavioral data from the participants who were shared with the previous experiment are the same as reported in the previous paper^5^. The participants were recruited from research cohorts at the University of California San Francisco (UCSF) Memory and Aging Center. All patients underwent a complete clinical evaluation and the diagnosis was made at a multidisciplinary consensus meeting for each patient individually. To make our cohort more uniform and representative of typical AD patients who are predominantly amnestic and dysexecutive in their symptom profile, we excluded the patients who fulfilled the current diagnostic criteria for atypical AD. The latter includes patients whose predominant deficits are in the language domain (logopenic variant of primary progressive aphasia) or in the visuospatial domain (posterior cortical atrophy syndrome)^24,25^. Fifteen out of 16 patients showed positive biomarkers for AD (Supplementary Table 1: Biomarkers and APOE genotypes of AD patients). One patient was not evaluated for biomarkers and showed a typical pattern of cortical grey matter loss on structural imaging. Eligibility criteria for age-matched healthy participants included normal cognitive performance, normal structural brain imaging, and absence of neurologic, psychiatric, and other major illnesses. All participants self-reported normal hearing and were assessed clinically for hearing loss. Each participant underwent a bilateral tone-hearing-test to verify the hearing status and confirmed proper earphone placement during the experiment. Informed written consent was obtained from all participants or their assigned surrogate decision makers. The study was approved by the UCSF institutional review board for human research and the methods were carried out in accordance with the relevant guidelines and regulations.

### Neuropsychological assessment

All participants (i.e. patients and controls) underwent Mini Mental State Examination (MMSE)^26^, and executive function testing of the cognitive battery (see below). In a structured caregiver interview, Clinical Dementia Rating scale (CDR), and CDR Sum of Boxes (CDR-SOB)^27^ were also determined for each patient. Statistical differences of demographic characteristics and neuropsychological test performance (i.e. MMSE and executive function) between patients and controls were examined using SAS (SAS 9.4, SAS Institute Inc.). The executive function tests used in this study included set shifting and cognitive control. Set shifting was assessed by modified trail-making-test^28^. The modified trail-making test requires the participant to draw lines linking items marked on paper and serially alternate between numbers and days of the week. The number of correct connections made within 120 seconds was recorded. To adjust for participants completing the task in less than 120 seconds, we calculated the dependent measure as the number of correct connections made per second. Cognitive control was assessed by the Stroop test^29,30^. There are two conditions for Stroop test. The control or color-naming task requires the participant to read blocks of text where the text and the ink color are matched (e.g., the word ‘red’ printed in red ink). The interference task requires the participant to name the color of text where the text and ink color are mismatched (e.g., the word ‘red’ printed in green ink). The number of correct responses in each condition is documented, and the cognitive control ability is estimated as the ratio of correct interference responses/color-naming responses. We estimated an executive composite score for each participant by averaging the Z-score values of set shifting and cognitive control performances. Z-score values for each participant were calculated based on age-matched normative control databases from the UCSF Memory and Aging Center.

### Experimental design and procedures

The pitch-perturbation experiment was completed in the MEG scanner with the participants lying in supine position. The MEG system (CTF, Coquitlam, British Columbia, Canada) consisted of 275 axial gradiometers and the data were recorded at a sampling rate of 1200 Hz. Three fiducial coils were placed on the nasion and left and right pre-auricular points to triangulate the position of the head relative to the MEG sensor array. The fiducial markers were later co-registered onto a structural magnetic resonance imaging scan to generate head shape^31^.

The experiment consisted of two successive 74-trial sessions. In each trial, the participant phonated the vowel /ɑ/ into a microphone while listening to the real-time audio feedback via headphones (Fig. 1B). In every trial, the pitch of the auditory feedback was perturbed for 400 ms following a randomly jittered delay of 200–500 ms from vocalization onset. The perturbation shifted the pitch of auditory feedback upwards or downwards by 100 cents (1/12^th^ of an octave). An equal number of pitch shifts that either raised or lowered the perceived pitch were pseudo-randomly distributed across the experiment. Each trial began with a visual cue (a clearly visible dot) presented on a screen directly in front of the participant. The participants were instructed to produce the vowel /ɑ/. Prior to the start of the experiment, the volume of auditory input through the earphones was adjusted to a comfortable level so that participants reported that their auditory feedback was the same as what they would normally hear when speaking. This was to ensure that the participants perceived the auditory feedback through their headphones as natural. The jittered perturbation onset prevented the participant from anticipating the timing of the perturbation while the pseudorandom selection of raising or lowering the pitch prevented the participant from anticipating the direction of the perturbation. The participants produced the vowel sound for the duration the visual cue displayed on the screen (~2.5 seconds), and then stopped phonation for the next 2.5 seconds during which time the screen was blank. After every 15 trials participants were cued for an optional break time.

Perturbation was accomplished by a digital signal processing (DSP) program running a real-time speech feedback alteration procedure. The input signal for DSP was the participant’s phonation, as picked up by an MEG-compatible optical microphone (Phone-Or Ltd., Or-Yehuda, Israel). The output from the DSP was fed back to the participant via MEG-compatible earplug earphones (model EAR-3A, Etymotic Research, Inc., Elk Grove Village, IL). The feedback alteration program was a vocoder process that recorded incoming speech and decomposed it into pitch and spectral envelope features^32,33^. The program then altered the pitch of the signal as it synthesized the outgoing speech signal. This process incurred a feedback delay of 12 ms. The data acquisition setup allowed us to simultaneously acquire MEG data, and several analog channels which included the microphone signal as well as an additional analog signal which showed the exact onset of the perturbation. The high signal-to-noise ratio in these analog channels enabled the easy identification of a threshold crossing to accurately mark the times of the voice onset and perturbation onset in the MEG data.

### Data processing and analysis

#### Audio data analysis

Raw audio data for each trial was first analyzed into time-course of pitch, using an autocorrelation-based pitch tracking method^34^. Trials with pitch tracking errors or incomplete utterances were excluded (50.7% and 14.8% for patients with AD and controls, respectively). In both AD patients and controls, among the trials that got excluded, about 50% were excluded due to incomplete utterances and the other 50% were excluded due to pitch tracking errors. However, exclusion of trials were based on criterion unrelated to the magnitude of the perturbation response and hence do not have any bearing on the analysis of included trials. We performed additional measures to verify the normality of our patient data and to ensure robust data analyses. An outlier detection analysis within the distribution of the percentages of excluded trials showed normal distribution across patients as determined by four different tests of normality (Shapiro-Wilk, P = 0.3584; Kolmogorov-Smirnov, P > 0.1500; Cramer-von Mises, P > 0.2500; Anderson-Darling, P > 0.2500), and with no outliers as determined by a Robust State Measures analysis in SAS. Next, we examined the distribution of each subject’s data to determine that they are normally distributed. This analysis identified three out of 16 AD patients and six out of the 13 control participants having skewed distributions. To ensure robust statistical analysis, we used a two‐tailed alpha‐trimming procedure in each of these participants with non-normal distributions to remove the extreme values that were greater than 2.5 SDs from their respective means. After the alpha trimming procedure, the percentage of total excluded trials were 51.2% and 16.7% for patients with AD and controls, respectively. The subsequent group statistics were performed on the alpha-trimmed data.

For each trial, an analysis interval of 1200 ms (−200–1000 ms from the perturbation-onset) was extracted, and the pitch changes were converted from hertz to cents relative to pre-perturbation baseline. For each participant, the pitch track for each trial was processed and expressed as deviations from the mean pitch track, averaged across all trials (i.e., up and down pitch-perturbations). Next, for each participant, responses to both upward and downward perturbations were combined into a single dataset depicting the absolute magnitude of the response (total number of trials per participant = combined trials of upward and downward perturbations for each participant). To generate the combined data set for each participant, the deviations from the mean time-course in response to the upward perturbations were first flipped (i.e., negate the cents deviation values of the time-course), and then the flipped trials were added to the data set of deviations from the mean time-course in response to the downward perturbations. The number of trials in the combined data set for each participant was equal to the number of trials in upward perturbations plus the number of trials in downward perturbations. To account for the trial-by-trial variability within participants and the variable number of analyzable trials (after excluding the trials with pitch tracking errors and incomplete utterances) between participants, we analyzed the behavioral data by combining all trials per subject in each group (total number of trials: AD = 965; controls = 1639), keeping the subject identity. We measured the peak behavioral response in each trial and compared the group differences using a linear mixed model analysis in SAS (PROC MIXED procedure), including the subject identification as a repeated measure.

#### MEG data analysis

The MEG sensor data were manually marked at the speech onset and at the perturbation onset. Third gradient noise correction filters were applied to the data and the data were corrected for a direct-current-offset based on the whole trial. Artifact rejection of abnormally large signals due to head movement, eye blinks, or saccades was first performed quantitatively by automated algorithms and then qualitatively by visual inspection. Sensor data was filtered at 120 Hz with a notch filter (4 Hz width).

We selected high-gamma band (65–150 Hz) response for the analysis of neural response as previous studies have reported that 65–150 Hz shows a reliable signal during the pitch perturbation response in human subjects^35^. Spatiotemporal estimates of neural sources of the induced high-gamma band activity were generated using a time–frequency optimized adaptive spatial filtering technique implemented in the Neurodynamic Utility Toolbox for MEG (NUTMEG; http://nutmeg.berkeley.edu). We used a variant of linearly constrained time frequency optimized minimum variance adaptive beamformers for the spatial filter as described in our prior publications^21,36,37^. Specifically, the spatial filter at each voxel was estimated from both the lead field matrix, the MEG data at each active window of interest, and MEG data at a corresponding baseline window. Importantly, the time frequency optimized spatial filter used distinct covariance matrices of the MEG data computed for every time frequency window of interest. The tomographic volume of source locations (voxels) was computed through an adaptive spatial filter (5 mm grid) that weights each location relative to the signal of the MEG sensors^36,38^. The source space reconstruction approach provided amplitude estimations at each voxel derived through the linear combination of spatial weighting matrix with the sensor data matrix^21,36,39^. A high-resolution structural MRI was obtained for each subject (see below) and was spatially normalized, with the resulting parameters being applied to each individual subject’s source space reconstruction through NUTMEG [standard Montreal Neurological Institute (MNI) template, statistical parametric mapping (SPM8) http://www.fil.ion.ucl.ac.uk/spm/software/spm8/].

Noise-corrected pseudo-F ratios were computed between the active windows (following the perturbation onset) and the pre-stimulus control baseline (preceding the onset of perturbation). We examined the period 100–300 ms post-perturbation-onset in 25 ms intervals. Group statistics were performed using statistical nonparametric mapping methods incorporated in the NUTMEG toolbox^38^. To minimize spatial frequency noise in the beamformer volumes, average and variance maps for each individual frequency band were calculated and smoothed using a Gaussian kernel with a width of 20 × 20 × 20 mm full-width-at-half-maximum^40^. High-gamma band MEG signals from deep brain structures have a low signal-to-noise ratio and therefore contribute to spatial blur and greater uncertainty of activity estimations. We restricted our analysis to the cortical surface by removing voxels corresponding to deep brain structures. We used a permutation test to create a null distribution from which the empirical p-values for the statistical contrast between groups were computed. Statistical significance was estimated by the significance of the test statistic (i.e. pseudo-F ratio) value from its position in this permuted distribution. To correct for multiple comparisons across space and time, we used 5% False Discovery Rate (FDR) in our analysis and thresholded the images with adjusted P values. Specifically, we determined the corrected P value threshold level at the 5% FDR cutoff level for all voxels that showed effects across the eight time-windows at the uncorrected (P < 0.05) threshold. The voxels that survived the FDR correction were then further thresholded using cluster correction in NUTMEG^21,38^ with a cutoff level of 30 voxels (clusters with more than 30 congruent voxels). Clusters in the thresholded statistical maps were discarded if they fell below the 95% of null-distribution cut-off following permutation testing and did not meet the required minimum value of 30 contiguous voxels. This approach minimized the possibility of observing spurious effects. The images were thresholded as such that only the voxels that exceeded the significance threshold can be seen in the figures.

#### Neural-behavioral correlation analysis

The group contrast between AD patients and controls demonstrated anatomic areas showing significantly increased and decreased high-gamma activity across the 100–300 ms post-perturbation time window. We identified the brain regions (see results, and Supplementary Table 2) that showed distinct group differences after applying the above described space and time varying multiple comparisons thresholds (Supplementary Table 2). To identify the neural-behavioral associations of these brain regions and the behavioral response, we used a general linear model (GLM) including the full cohort of patients and controls in which the predictor variables included the high-gamma band activity of each of the eight ROIs (for each subject), and the group identity (patient vs. control). The dependent variable was the peak behavioral response. This analysis identified two ROIs as significant predictors of the model, namely left prefrontal and right middle temporal regions. Next, we used an extended model of GLM including the interaction between group identity and the neural responses of left prefrontal and right middle temporal regions in addition to the previously entered variables. In our previous behavioral experiment, we found that the peak behavioral response was significantly correlated with executive function abilities. To examine the relationship between neural activity and peak behavioral response above and beyond the associations of executive abilities we further included the composite executive function score into the extended GLM model.

Next, we examined the associations between the peak behavioral response and the high-gamma band time series activity within each of the 25 ms time windows for the two ROIs, identified from the GLM. To this end we employed an analysis of covariance (ANCOVA) model including the full cohort of patients and controls in which the predictor variables included the peak behavioral response in each subject and the group identity (patient vs. control). The dependent variable was the mean high gamma power of each ROI, namely, left prefrontal cortex and right middle temporal region. A separate ANCOVA model was used for each ROI. We examined the associations between the peak behavioral response and the high-gamma band activity within each of the 25 ms time windows for these two ROIs, using identical ANCOVA models. The left prefrontal cortex activity was evaluated during the 100–250 ms post perturbation onset while the right middle temporal region activity was evaluated during the 200–300 ms post-perturbation time windows. We examined the relationships between peak behavior and the neural activity of left prefrontal and right middle temporal regions, first averaged across each of their respective time durations; next, within 25 ms bins.

#### Magnetic resonance image acquisition

Structural MRI images were acquired on a 3-Tesla Siemens MRI scanner at the Neuroscience Imaging Center-UCSF for 15 of the 16 AD patients, and for all control participants. The remaining 1 patient was evaluated with an MRI scan obtained at an outside facility within 2 years of the MEG evaluation. The structural MRIs were used to generate head models for source space reconstruction of the MEG sensor data.

## Results

### Clinical and demographic characteristics of participants

Patients with AD were mild to moderately impaired with a mean CDR of 0.84 ± 0.24 (n = 5 patients with CDR of 0.5, n = 11 patients with CDR of 1) and a mean MMSE of 21.50 ± 3.44 (Table 1). Control participants were matched with AD patients in age, sex, handedness and race, yet showed a significantly higher number of years in education than patients (Table 1). Patients with AD were 1.56 standard deviations below the executive composite scores derived from age-matched normative control populations at the UCSF Memory and Aging Center.

**Table 1:** Participant demographics.

	Controls (n = 13)	AD Patients (n = 16)	P Value^¶^
Age – yr	63.70 ± 5.54	60.02 ± 9.84	0.240
Female sex – no. (%)	10 (76.92)	11 (68.75)	0.624
White – no. (%)^†^	12 (100.00)	14 (100.00)	1.000
Education – yr	17.69 ± 1.60	15.63 ± 2.83	0.023
Right handedness – no. (%)	13 (100.00)	14 (87.50)	0.187
MMSE^‡^	29.77 ± 0.44	21.5 ± 3.44	<0.0001
CDR	0 ± 0	0.84 ± 0.24	<0.0001
CDR-SOB	0 ± 0	4.91 ± 1.19	<0.0001

Abbreviations: AD = Alzheimer’s disease; CDR = Clinical Dementia Rating; CDR-SOB = CDR Sum of Boxes; MMSE = Mini-Mental State examination.

The AD cohort included five patients with CDR = 0.5 and 11 patients with CDR = 1; Values for age are means ± SD. Age ranges are 48.99–84.32 and 56.22–75.56, for Alzheimer’s disease patients and control participants respectively.

^**¶**^Statistical tests were unpaired *t*-test for age, education, MMSE; Fisher Exact test for sex, race, and handedness.

^†^Race was self-reported; one control participant and 2 patients with AD withheld from reporting race.

^‡^Scores on the MMSE range from 0 to 30, with higher scores denoting better cognitive function.

### Patients with AD showed an elevated behavioral pitch perturbation response compared to age-matched controls

In a previous study we reported the behavioral response to perturbed pitch of the auditory feedback during speaking in patients with AD and age-matched controls (Ranasinghe *et al*., 2017). The behavioral results in the current study, which included a partially overlapping participant cohort from the previous study, were similar. In brief, the behavioral response to pitch-perturbations in auditory feedback in both patients and controls was compensatory—opposite to the direction of perturbation. When time locked to the perturbation onset both groups started responding around 200 ms post-perturbation-onset (Fig. 1C) and reached a peak response around ~500 ms post-perturbation-onset (peak latency: controls (Mean ± SE) = 487 ± 7.0 ms; patients (Mean ± SE) = 485 ± 9.9 ms; linear mixed model, F(1) = 0.03, P = 0.87). The average peak behavioral response of control participants in the current experiments was 18.4 ± 0.75 cents (Mean ± SE) and was consistent with the responses recorded from younger normal controls in previous studies^5,21,41^. Patients with AD, in contrast, showed a significantly elevated peak behavioral response averaging at 24.3 ± 1.4 cents (Mean ± SE; linear mixed model F (1) = 16.05, P < 0.0001; Fig. 1C).

### Patients with AD showed distinctive changes of high-gamma activity during pitch perturbation response

**Figure 2:**
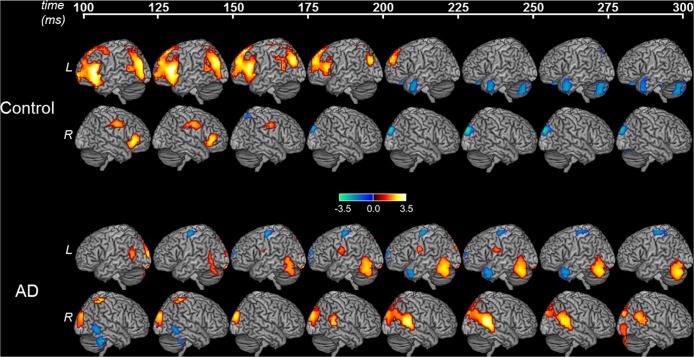
Cortical responses during pitch perturbation. MEGI derived high-gamma activity (65–150 Hz) aligned to perturbation onset, in 25 ms intervals, for controls AD patients. The color maps indicate differences in high-gamma power as compared to pre-perturbation baseline in each group. Color scale represents t-values. Hot colors indicate higher activity, and cold colors indicate lower activity, compared to baseline. The controls show robust responses in frontal cortex and posterior parietal cortex, especially during the early part of the response (i.e. 100–200 ms post-perturbation-onset). Patients with AD, in contrast, show attenuated responses in frontal and posterior parietal cortices. Abbreviations: AD = Alzheimer’s disease; L = left hemisphere; MEGI = magnetoencephalographic imaging; R = right hemisphere.

Neural activity preceding the onset of behavioral response presumably involves the detection of the feedback error and preparation for the corrected motor response. During the 100–200 ms post-perturbation-onset, age-matched normal controls showed robust elevations of high-gamma band activity over the left prefrontal and left posterior parietal cortices (Fig. 2). High-gamma activity was also evident in the right prefrontal and parietal cortices during 100–175 ms from perturbation onset in the control group (Fig. 2, Control response). After 200 ms from perturbation onset, high-gamma activity diminished in controls. During 200–300 ms post-perturbation-onset, the high-gamma activity in the left anterior temporal lobe and right posterior parietal cortex were significantly below the baseline in the controls (Fig. 2, Control response). Collectively, these results are consistent with a strong involvement of higher order auditory regions, premotor and frontal cortices in detection and correction for auditory feedback errors, as predicted by current models^4^.

Patients with AD, in contrast, showed an attenuated response over the higher order auditory regions and virtually absent high-gamma activity in frontal cortex, during 100–200 ms post-perturbation-onset (Fig. 2, AD response). Instead, AD patients showed a significantly elevated high-gamma activity over posterior parietal and occipital regions in both the left and right hemispheres (Fig. 2, AD response). Furthermore, such posterior dominant activity patterns were consistent throughout the duration of 100–300 ms post-perturbation-onset and showed an evolving increase over the time course until ~275 ms post-perturbation-onset. Starting from 175–200 ms post-perturbation-onset, there was also a progressively increasing high-gamma activity in the right posterior middle temporal cortex. High-gamma activity was also seen in the left ventral motor cortex between 175–250 ms. AD patients also showed reduced high-gamma activity over the left superior precentral regions throughout the duration (Fig. 2). The only consistent pattern in both controls and patients was the reduced left anterior temporal lobe activity during 200–275 ms post-perturbation-onset.

**Figure 3:**
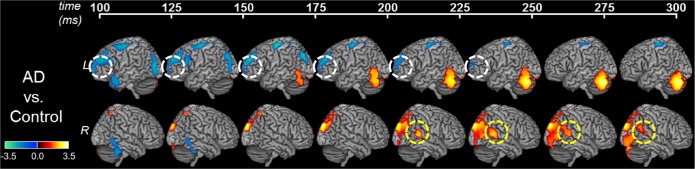
The altered patterns of high-gamma activity in patients with AD during perturbation. The images show the direct comparison of MEGI derived high-gamma activity (65–150 Hz) between patients with AD and controls. Specifically, the patterns indicate the activity in patients after subtracting the activity of controls, time locked to perturbation onset in 25 ms intervals. Color scale represents t-values. Hot colors indicate higher activity, and cold colors indicate lower activity, compared to controls. Statistical models identified the left prefrontal (white dotted circles) region and right middle temporal region (yellow dotted circles) activity as significantly associated with the peak behavioral response in pitch perturbation response. Abbreviations: AD = Alzheimer’s disease; L = left hemisphere; MEGI = magnetoencephalographic imaging; R = right hemisphere.

A direct comparison between patients and controls clearly illustrated the altered patterns of high-gamma activity patterns in patients with AD (Fig. 3, AD vs. control). The most salient differences found in AD patients included: reduced high-gamma activity in the left prefrontal, and occipital cortices during early response (i.e. 100–250 ms post-perturbation-onset); increased high-gamma activity in the right posterior middle temporal cortex during late response (i.e. 200–300 ms post-perturbation-onset); increased high-gamma activity in the right posterior parietal and bilateral occipital cortices during late response (i.e. 175–300 ms post-perturbation-onset); and decreased high-gamma activity in the left superior-precentral regions, throughout (100–300 ms post-perturbation-onset).

### Left prefrontal and right middle temporal high-gamma activity reveal neural correlates of pitch perturbation response

We next sought to investigate the specific neural underpinnings of the elevated pitch perturbation response in AD patients. We identified eight regions-of-interest (ROIs) that showed statistically significant differences between patients and controls (Supplementary Table 1). To examine the specific contributions of the activity patterns in each of the eight ROIs in predicting the peak behavioral response, we used a GLM model with the peak behavioral response as the dependent variable, while the activity patterns within each of the eight ROIs and group identity were predictor variables. The overall model explained 61.3% of the variance of the peak behavior (model-R^2^ = 61.3; F = 2.45; P = 0.047) and identified two ROIs as statistically significant predictors of peak behavioral response. These included the left prefrontal cortex (Fig. 3, white dotted circles), and right middle temporal cortex (Fig. 3, yellow dotted circles), which independently predicted the degree of peak behavior (Left prefrontal: F = 7.96, P = 0.012; right middle temporal: F = 6.79, P = 0.018). Specifically, lower activity in the left prefrontal cortex predicted higher peak behavioral response (Beta = −313.53 ± 124.06), whereas higher activity in the right middle temporal region predicted higher peak behavioral response (Beta = 98.14 ± 79.27). In an extended GLM model we included the group-by-ROI interaction effects into the model with relation to left prefrontal and right middle temporal regions and also a composite score of executive function abilities. The extended model identified left prefrontal and right middle temporal regions as significant predictors of peak behavioral response, even after inclusion of the aforementioned additional covariates. Moreover, there were no significant group-by-ROI interaction effects (Group-by-left prefrontal: F = 0.06; P = 0.80; Group-by-right middle temporal: F = 0.78, P = 0.39). Both models further revealed that there was no significant main effect of group identity towards this association (F = 1.96; P = 0.18).

**Figure 4:**
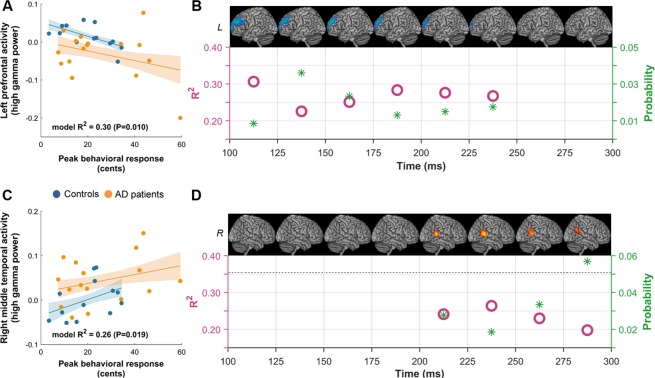
Left prefrontal activity and right middle temporal activity predict peak behavioral response in pitch perturbation response. (**A**) In an analysis of covariance model (ANCOVA) on the combined cohort of both patients and controls, the average high-gamma activity of left prefrontal cortex across the 100–250 ms post-perturbation-onset was significantly negatively correlated with the peak behavioral response. (**B**) The R^2^ of the model predictions (pink circles; left-side Y axis) and the p-values (green stars; right-side Y axis) of the association between left prefrontal activity and peak behavioral response in each 25 ms window. (**C**) The average high-gamma activity of the right posterior middle temporal cortex across the 200–300 ms post-perturbation-onset was significantly positively correlated with the peak behavioral response. (**D**) The R^2^ of the model predictions (pink circles; left-side Y axis) and the p-values (green stars; right-side Y axis) of the association between right middle temporal activity and peak behavioral response in each 25 ms window. Abbreviations: AD = Alzheimer’s disease; L = Left hemisphere.

An ANCOVA model with the main effects of peak behavioral response and group identity as predictor variables revealed significant associations with the left prefrontal cortex activity averaged across 100–250 ms post-perturbation-onset (Fig. 4A; model-F = 5.52, model-P = 0.010, model-R^2^ = 0.30). The results further showed that lower activity in the left prefrontal cortex was predicted by higher peak behavioral response (F = 4.58, P = 0.042). An extended ANCOVA model with interaction between peak behavioral response and group identity included as predictor variables in addition to their main effects, showed no significant interaction between the peak behavioral response and the group identity (interaction-F = 0.13, interaction-P = 0.723; model-F = 3.60, model-P = 0.027). This finding demonstrated that the slopes between patients and controls are not significantly different from each other indicating the homogeneity of slopes between the two groups, and thereby strengthened the design of the ANCOVA model on the combined data set. Similar simple ANCOVA models (models without interaction) at each 25 ms window of 100 to 250 ms post perturbation time period showed significant associations between left prefrontal high gamma band activity and peak behavioral response (Fig. 4B).

A separate analysis based on identical ANCOVA models was used to examine the neural-behavioral associations of the right middle temporal region showed significant positive associations with the activity averaged across 200–300 ms post-perturbation-onset (Fig. 4C; model-F = 4.60, model-P = 0.019, model-R^2^ = 0.26). Specifically, higher peak behavioral response showed a trend for a positive association with the degree of right middle temporal region (F = 3.5, P = 0.072). Similar to the findings in the left prefrontal cortex, a full ANCOVA model with the interaction between peak behavioral response and group identity included as predictors showed no significant interaction between the peak behavioral response and the group identity, indicating the homogeneity of slopes for patients and controls (interaction-F = 0.28, interaction-P = 0.601; model-F = 3.08, model-P = 0.046). ANCOVA models for each 25 ms interval during 200–250 ms interval showed significant neural behavioral relationships (Fig. 4D).

Collectively, our results indicate that reduced high gamma band activity in the left prefrontal region during early phase of perturbation (100–250 ms post perturbation onset) and increased high gamma band activity in the right middle temporal region during the late phase of the perturbation (200–300 ms post-perturbation onset) are significant neural correlates of a higher peak behavioral response in pitch perturbation response.

## Discussion

In this study we examined the neural correlates of abnormal sensorimotor integration processes in AD. In response to perturbed pitch in the auditory feedback while speaking, AD patients demonstrated distinct high-gamma band network activity when compared to age-matched controls. In particular, AD patients showed reduced high-gamma activity in the left prefrontal cortex and increased high-gamma activity in the right posterior middle temporal cortex. Importantly the impaired left prefrontal activity occurred early, followed by the increased right middle temporal activity, demonstrating the temporal evolution of speech-motor control activity. Reduced prefrontal activation and enhanced recruitment of right temporal cortices were significant contributors to abnormally enhanced pitch perturbation response, in AD patients. These results signify potential neural substrates of disrupted cortico-cortical connections in neural networks in AD.

Growing evidence from advanced neuroimaging techniques strongly supports the notion that the underlying architecture of cognitive function consists of dynamically interactive neural networks^42,43^. This understanding is a clear shift from an earlier view where each cognitive process was localized to a specific anatomic region in the brain. Indeed, the anatomic involvements of neurodegenerative diseases, of which the cardinal manifestations are progressive loss of cognitive abilities, have been directly mapped onto large-scale neural networks in the brain^13,44^. In this context, quantification of network dysfunctions and their behavioral correlates are important markers of neurodegenerative disease processes^45^. The current results demonstrate that speech-motor control network activity reliably yields neural as well as behavioral measures of network dysfunction in AD. Because pitch perturbation response is involuntary^46^, it has the added advantage of minimizing the confounding behavioral effects of cognitive deficits on the task performance, in patients with AD. As such, the neural and behavioral correlates of pitch perturbation response could potentially provide useful scales of network integrity to gauge disease progression and therapeutic efficacy in clinical trials of AD^47^.

AD is considered as primarily affecting the higher level cognitive functions such as executive, memory, visuospatial and language^48^. However, the presence of an abnormal pitch perturbation response demonstrates that patients with AD also have significant abnormalities in low-level sensorimotor integration processes. It is conceivable that aberrant neural integration mechanisms may be shared across neural circuits responsible for sensorimotor integration and higher cognitive processes and may occur either at network level or at molecular level. At network-level, a common anatomic region may serve as a critical hub in separate networks. At molecular-level, the same pathophysiological cellular and molecular processes may affect neural circuitry in separate networks. The neurobehavioral correlates of the current study demonstrate strong associations between left prefrontal cortex activity and speech-motor-control network output. Left prefrontal cortex has been identified as a key anatomic region involved in executive and cognitive control behaviors^49,50^. It is therefore likely that left prefrontal cortex acts as a common hub for both circuits—speech-motor control and executive function, and hence a mutual target of network disruption in AD. The current results, however, do not exclude the possibility of shared pathophysiological cellular and molecular processes affecting distinct networks. For example, when comparing AD patients and age-matched controls we also found that other brain regions including the dorsal parietal and occipital cortices also show significantly reduced neural activity patterns, although these regions did not show significant neurobehavioral correlations with the pitch perturbation response. As such, it is still a possibility that shared cellular level abnormalities associated with AD pathophysiology may give rise to the abnormal activity reductions in these additional regions, although they are not necessarily recruited by the speech motor control network during the task.

 Increased activity patterns in patients with AD compared to controls have been demonstrated in task engaged brain networks, and also in resting brain networks based on FDG-PET imaging^51,52^. Such evidence has supported the hypothesis that additional neural resources are recruited to achieve behavioral goals in AD. Consistent with these observations we found that AD patients showed increased neural activity over the right posterior middle temporal cortex compared to age-matched controls and that these patterns showed a significant trend in its associations with peak behavioral response in pitch perturbation paradigm. However, the overall behavioral outcome in AD patients as demonstrated in our previous experiment and shown again in the current experiment, is abnormally increased compared to age-matched elderly participants, indicating that these additional neural resources fall short of achieving the optimal behavioral target. Collectively, these findings would suggest that additional brain regions with enhanced activity may represent various neural processes involved in AD brains that mediate the behavioral goals yet give little indication of how successfully these processes are able to support the projected outcome.

Several previous experiments reported an elevated behavioral response to pitch feedback perturbations in patients with other neurological disorders, such as Parkinson’s disease (PD)^53–56^, and cerebellar degeneration (CD)^57^. The abnormal sensorimotor integration in PD has been linked to impaired feedforward signals delivered by basal ganglia, the latter region being a major target of the pathophysiological processes in PD. Likewise, the abnormal feedback response in CD has been linked to impaired feedforward signals from cerebellum. In both conditions, it is hypothesized that the balance between sensory feedback and motor feedforward processes in the speech motor control network is biased towards sensory feedback. The increased reliance on auditory feedback may increase the sensitivity to perturbations thus leading to an enhanced behavioral pitch perturbation response. Simultaneous EEG recordings have demonstrated larger event related potentials indicating enhanced activity in the inferior frontal gyrus, precentral gyrus, postcentral gyrus, and middle temporal gyrus associated with the enhanced behavioral response in PD patients^53^. Taken together with the results from the current study it is reasonable to infer that distinct underlying mechanisms may subserve enhanced pitch perturbation response in different disease processes.

The current study is also the first to report brain activity dynamics during the pitch perturbation response in healthy older adults. Compared to previous reports in healthy young subjects^21,41^, one striking difference in healthy older adults is the significant involvement of left prefrontal cortex during the early phase of response. Despite these neural differences, both old and young subjects showed a similar degree of behavioral response to altered pitch in their auditory feedback (18.62 cents vs. 18.98 cents per 100 cents perturbation, in old and young healthy subjects, respectively)^21^. These results are consistent with previous reports on other psychophysical tasks showing that young and old participants reach the same behavioral performance, albeit with distinct patterns of neural activity. For example fMRI experiments based on visual object processing^58,59^, visual and verbal memory tasks^60^, and on cognitive control^61^, have demonstrated that both young and old individuals achieve the same behavioral accuracy although the older individuals generate neural activity patterns that are distinctly different from the younger subjects. Indeed, a consistent finding in these experiments was the comparatively stronger prefrontal activity in the older subjects^62^. Models of cognitive aging have explained the age dependent hyperactivity as a mechanism compensating for functional declines elsewhere or for increased ‘noise’ generated by the reduced precision of receptive field properties^63^. Although, the current results do not distinguish between these explanations they certainly provide evidence for functional reorganization of the left prefrontal regions supporting cognitive abilities of the aging brain. Moreover, our results further demonstrated that patients with AD have significantly impaired left prefrontal cortex activity compared to healthy older individuals indicating deficits in neural substrates of cognitive reserve in AD patients^64^.

Our study is not without limitations. First, the neural activity patterns we recorded contain both the auditory afferent as well as the motor efferent processes^4^. Studying neural responses for passive listening to audio feedback will allow us to separate out these phenomena. Future studies comparing these individual effects between patients with AD and older controls will enlighten us on disease specific manifestations of auditory feedback encoding and motor command processing. Second, the current study was primarily designed to understand the differences between patients with AD and healthy older subjects. As such, we did not present a comprehensive analysis of the age-related changes in the sensorimotor integration of pitch perturbation response by comparing healthy old and young participants. A future study comparing old and young would allow us to understand the specific age-related changes. Third, we identified the anatomic regions in relation to neurobehavioral correlations by subtracting the activity between controls and AD patients. This methodology was based on the assumption that both healthy older subjects and AD patients will recruit similar anatomic regions albeit at different levels of activity. Although definite validation of this assumption would come from significant neurobehavioral correlations within each group itself, the sample sizes of the current cohorts were underpowered to test effects within each diagnostic group separately. Nonetheless, the significant findings in the ANCOVA analysis in the combined sample of patients and controls supported this assumption.

## Supplementary information

Supplement

## Data Availability

Anonymized data will be shared on request from qualified investigators for the purposes of replicating procedures and results, and for other non-commercial research purposes within the limits of participants’ consent.
