# Supplementary material for: Neural correlates of abnormal auditory feedback processing during speech production in Alzheimer’s disease
Source: Sci Rep. 2019 Apr 5;9:5686. doi: 10.1038/s41598-019-41794-x (PMC6450891; doi:10.1038/s41598-019-41794-x)
Supplement: Supplementary file 1 — Supplement [file 41598_2019_41794_MOESM1_ESM.pdf]

SUPPLEMENT

Neural correlates of abnormal auditory feedback processing during speech production in Alzheimer's disease

Kamalini G Ranasinghe*[,a], Hardik Kothare[b,c,d], Naomi Kort[b,c], Leighton B. Hinkley[b,c], Alexander J Beagle[a], Danielle Mizuiri[c], Susanne M Honma[c], Richard Lee[a], Bruce L Miller[a], Maria Luisa Gorno-Tempini[a], Keith A Vossel[a,e], John F Houde[b,c] , Srikantan S Nagarajan[b,c],

[a]Memory and Aging Center, Department of Neurology, University of California San Francisco, San Francisco, CA 94158

[b]Speech Neuroscience Laboratory, Department of Otolaryngology - Head and Neck Surgery, University of California San Francisco, San Francisco, CA 94143

[c]Biomagnetic Imaging Laboratory, Department of Radiology and Biomedical Imaging, University of California San Francisco, San Francisco, CA 94143

[d]Department of Bioengineering, The UC Berkeley - UCSF Graduate Program in Bioengineering

[e]N. Bud Grossman Center for Memory Research and Care, Institute for Translational Neuroscience, and Department of Neurology, University of Minnesota, Minneapolis, MN 55455

*Corresponding author: Kamalini G Ranasinghe, MD PhD
Postdoctoral fellow
Memory and Aging Center, Department of Neurology
University of California San Francisco
675 Nelson Rising Lane, Suite 190, San Francisco, CA 94158-1207
Tel: 415-514-8847
e-mail: Kamalini.ranasinghe@ucsf.edu

SUPPLEMENT

The supplement includes:

1. **Supplementary Table 1:** Biomarkers and APOE genotypes of Alzheimer's disease patients

2. **Supplementary Table 2:** Anatomic regions showing distinctive activity in Alzheimer's disease patients vs. controls.

1. **Supplementary Table 1:** Biomarkers and *APOE* genotypes of Alzheimer's disease patients

Patient number	Amyloid Imaging*	CSF Biomarkers†	Brain MRI	APOE Genotype
1	Positive	-	L > R hippocampal atrophy and diffuse cerebral atrophy with posterior predominance	ε4/ε4
2	Positive	-	Diffuse atrophy predominantly in the hippocampi and posterior cortex	ε3/ε4
3	Positive	-	R > L parietal atrophy	ε2/ε3
4	Positive	-	Bilateral parietal atrophy	-
5	Positive	-	Bilateral hippocampal atrophy and diffuse cortical atrophy	ε3/ε3
6	Positive	-	Bilateral parietal atrophy	-
7	Positive	-	Bilateral hippocampal atrophy and diffuse cortical atrophy	-
8	Positive	-	Bilateral parietal atrophy	ε3/ε3
9	Positive	-	R > L parietal atrophy	-
10	-	Aβ42=125.0<br>t-Tau=559.4<br>p-Tau= 82.0<br>Aβ42-Tau Index=0.14	Bilateral hippocampal atrophy	ε3/ε4
11	-	Aβ42=399.5<br>t-Tau=527.6<br>p-Tau=70.5<br>Aβ42-Tau Index=0.46	Bilateral hippocampal atrophy and L > R parietal atrophy	ε3/ε4
12	-	Aβ42=230.0<br>t-Tau=293.5<br>p-Tau=54.2<br>Aβ42-Tau Index=0.39	Bilateral hippocampal and L > R parietal atrophy	ε3/ε4
13	-	Aβ42=473.7<br>t-Tau=366.6<br>p-Tau=74.75<br>Aβ42-Tau Index=0.70	Bilateral hippocampal atrophy and parietal atrophy	-
14	-	Aβ42=240.3<br>t-Tau=1090.5<br>p-Tau=142.1<br>Aβ42-Tau Index=0.16	Bilateral hippocampal atrophy and parietal atrophy	-
15‡	-	-	Bilateral hippocampal atrophy and diffuse cortical atrophy with posterior predominance	ε3/ε3

Patient number	Amyloid Imaging*	CSF Biomarkers†	Brain MRI	APOE Genotype
16	-	-	Bilateral hippocampal atrophy	-

Abbreviations: Aβ42 = amyloid-β peptide ending in amino acid residue 42; CSF = cerebrospinal fluid; L = left; MRI = magnetic resonance imaging; p-Tau = tau phosphorylated at threonine 181; R = right; t-Tau = total tau.

* Positron emission tomography agent was ^{11}C-Pittsburgh compound B for patients 1-4 and ^{18}F-AV-45 for patients 5-9.

† Units for Aβ42, t-Tau, and p-Tau are pg/ml; Values supporting a diagnosis of Alzheimer's disease include: p-Tau level >61 pg/ml and Aβ42-Tau Index <1.0 (Athena Diagnostics).

‡ Alzheimer's disease was confirmed by autopsy according to National Institute on Aging–Reagan Institute criteria.

2. **Supplementary Table 2:** Anatomic regions showing distinctive activity in Alzheimer's disease patients vs. controls.

	Anatomic area	MNI coordinates of the peak voxel†	Duration of activity (time from post perturbation onset)
Left Hemisphere	Left prefrontal (BA 10)*	-5 65 5	100 - 250
	Left precentral (BA 6)*	-40 -5 60	100 - 300
	Left middle occipital (BA 19)*	-45 -85 15	100 - 175
	Left calcarine (BA 18)	-5 -90 -10	175 - 300
	Left inferior occipital	-45 -80 -15	150 - 300
	Left anterior temporal (BA 38)* ‡	-50 10 -35	100 - 125
Right hemisphere	Right middle temporal (BA 37)	65 -55 10	200 - 300
	Right superior parietal (BA 7)	15 -65 60	100 - 300
	Right precuneus (BA 7)	5 -80 50	125 - 300
	Right posterior inferior temporal*‡	35 -35 -20	100 -150

Abbreviations: BA = Brodmann area; MNI coordinates = Montreal Neurological Institute brain coordinates.

* Indicates the regions where patients showed reduced high-gamma-band activity patterns compared to controls.

† The MNI coordinates represent the peak voxel within the cluster of voxels identified.

‡ Left anterior temporal and right posterior inferior temporal regions were not included into the ANCOVA analysis since the effects were very transient (<50ms). The ANCOVA analysis included the rest of the eight ROIs.
